# Removal of the Superior Maxilla on the Left Side, with a Large Tumor Involving That Bone

**Published:** 1853-07

**Authors:** 


### ARTICLE X.

*Removal of the Superior Maxilla on the left side, with a large Tumor involving that Bone.* Under the care of Mr. HANCOCK.

THE operating theatre of this hospital was crowded on Monday last, 5th June, as it was understood that Mr. Hancock would operate upon a little girl about six years old, whose case had excited a very lively interest. The child presented a very sad appearance, as the whole of the left side of the face was occupied by a tumor about the size of half a fetus' head, which by its projection outwards and upwards had completely closed the left eye, flattened the nose, and drawn the mouth, in such a manner that these organs had entirely lost their primary form. The tumor was hard and nodulated, not very painful, and had somewhat encroached upon the cavity of the mouth. The latter aperture could, however, be opened and shut at will. A feature of much interest was the surprising rapidity with which the tumor had grown, only a few months having elapsed since it had first been noticed. This circumstance naturally led to the supposition that the growth might be of a malignant nature, as it is but seldom that fibrous or purely bony tumors make such rapid progress. Whatever the nature of the growth, it was plain that the case was of such a kind that the only chance of saving the child's life was to remove the unsightly mass. In fact, it would appear that the poor little girl had been sent from the country to Mr. Hancock for the purpose.

Chloroform was administered by means of a piece of lint sprinkled with the narcotizing agent; and though the effect was not kept up all through the operation, it lulled the child considerably, and probably saved the little patient a great amount of pain. Mr. Hancock made the first incision in the median line, or rather between the nostrils, down through the whole thickness of the upper lip, as the nose was pressed by the tumor beyond the mesial line towards the right side; the second through the centre of the tumor from above downwards; and the third from the outer canthus of the eye in the same direction, a wedge-shaped piece of skin being left attached to the tumor. The knife was then run from the root of the nose to the zygomatic arch close under the border of the lower lid. By now detaching the skin from over the tumor from these lines of incision, the whole mass was soon laid bare. Mr. Hancock now divided the zygomatic process with strong bone forceps; one of the incisor teeth was pulled out, and the ascending process of the superior maxilla, as well as the whole depth of the hard palate, successively divided with the same instrument. The tumor, which now projected spherically to the size of a large orange, was now attached only by the orbital plate and the deep-seated parts. The anterior portion of the orbital plate was easily cut through, and the forceps being applied towards the posterior attachments of the antrum and hard palate, the whole mass, by using a little force, was completely detached and removed. The bones yielded everywhere to a moderate cutting force, and seemed to have undergone some softening.

The cavity now left was of course considerable, but the hemorrhage was not so copious as it usually is in these operations. In fact there was hardly any bleeding after the mass had been removed, the internal maxillary and other smaller branches being probably clogged up by the pressure of the tumor. No actual cautery, nor any other hæmostatic means, were required, and the child was able to swallow a little wine which was put towards the pharynx. It was now found that the skin had been so stretched by the tumor, that notwithstanding some had been left on the growth, there was abundance of integument. The lines of incision were carefully brought together, and kept in apposition by numerous interrupted sutures, the child in the mean time giving very little trouble, and bearing the manipulations very patiently. The whole of the left side of the face rose and fell like a valve at each respiratory act, but the features had already lost the former repulsive aspect.

The tumor seemed to be, instead of an encephaloid growth, as was at first expected, of a bony character, judging from its weight. The parts which could be recognised, were the hard palate with its teeth and alveoli, one of the turbinated bones, the nasal duct gliding by the side of it, and part of the zygomatic process running into the malar bone, which was completely involved in the tumor. The latter seemed, in fact, formed of an osseous expansion of the antrum, a state of parts which will probably be found when the section is made.

Mr. Hancock, in addressing the pupils, remarked that this tumor had grown very rapidly, as it was only last November that a surgeon in the country had been consulted on the case, there having been pain and congestion in the part. Already, at that time, however, the nature of the disease did not escape that gentleman, and it was soon afterwards found that the child would have to be sent to Mr. Hancock for operation. The nodules which had existed on the tumor resulted from some parts within giving way, and very alarming hemorrhage had then taken place. Mr. Hancock further stated, that he had never known the ascending process of the superior maxilla to assume such thickness as it had done in this case, it being now fully an inch and a half wide. He had reserved the division of the hard palate, and the parts around, for the last step of the operation, as this division sometimes gives rise to much hemorrhage, which might cause suffocation. He had good hopes of this case, and trusted it would end as successfully as one of the same kind which had been operated upon by him in this theatre some time ago. The patient was a young man, likewise affected with a bony tumor of the antrum, and had done extremely well after the removal of the superior maxilla.

We shall watch this case with much interest, and acquaint our readers with the results.—*London Lancet.*

